# Contribution of low population immunity to the severe Omicron BA.2 outbreak in Hong Kong

**DOI:** 10.1038/s41467-022-31395-0

**Published:** 2022-06-24

**Authors:** Lin-Lei Chen, Syed Muhammad Umer Abdullah, Wan-Mui Chan, Brian Pui-Chun Chan, Jonathan Daniel Ip, Allen Wing-Ho Chu, Lu Lu, Xiaojuan Zhang, Yan Zhao, Vivien Wai-Man Chuang, Albert Ka-Wing Au, Vincent Chi-Chung Cheng, Siddharth Sridhar, Kwok-Yung Yuen, Ivan Fan-Ngai Hung, Kwok-Hung Chan, Kelvin Kai-Wang To

**Affiliations:** 1 State Key Laboratory for Emerging Infectious Diseases, Carol Yu Centre for Infection, Department of Microbiology, School of Clinical Medicine, Li Ka Shing Faculty of Medicine, Pokfulam, The University of Hong Kong, Hong Kong SAR, China; 2 Quality and Safety Division, Hospital Authority, Hong Kong SAR, China; 3 Centre for Health Protection, Department of Health, Hong Kong SAR, China; 4 Department of Microbiology, Queen Mary Hospital, Pokfulam, Hong Kong SAR, China; 5 Infection Control Team, Queen Mary Hospital, Hong Kong West Cluster, Hong Kong SAR, China; 6 Department of Clinical Microbiology and Infection Control, The University of Hong Kong-Shenzhen Hospital, Shenzhen, China; 7 Centre for Virology, Vaccinology and Therapeutics, Hong Kong Science and Technology Park, Hong Kong SAR, China; 8 Department of Medicine, School of Clinical Medicine, Li Ka Shing Faculty of Medicine, Pokfulam, The University of Hong Kong, Hong Kong SAR, China

**Keywords:** Viral infection, SARS-CoV-2, Epidemiology, Phylogeny

## Abstract

Monitoring population protective immunity against SARS-CoV-2 variants is critical for risk assessment. We hypothesize that Hong Kong’s explosive Omicron BA.2 outbreak in early 2022 could be explained by low herd immunity. Our seroprevalence study using sera collected from January to December 2021 shows a very low prevalence of neutralizing antibodies (NAb) against ancestral virus among older adults. The age group-specific prevalence of NAb generally correlates with the vaccination uptake rate, but older adults have a much lower NAb seropositive rate than vaccination uptake rate. For all age groups, the seroprevalence of NAb against Omicron variant is much lower than that against the ancestral virus. Our study suggests that this BA.2 outbreak and the exceptionally high case-fatality rate in the ≥80 year-old age group (9.2%) could be attributed to the lack of protective immunity in the population, especially among the vulnerable older adults, and that ongoing sero-surveillance is essential.

## Abstract (web summary)

Hong Kong experienced a severe wave of SARS-CoV-2 in early 2022. Here, the authors use genomic and serosurveillance data and show that this wave was dominated by the Omicron BA.2 sublineage, and that low protective immunity, particularly in older age groups, contributed to its severity.

## Introduction

Severe acute respiratory syndrome coronavirus 2 (SARS-CoV-2), the causative agent of COVID-19, is characterized by efficient person-to-person transmission, propensity to cause severe disease, and frequent emergence of variants that escape population immunity^1–3^. The rapid surge of severe cases which require hospitalization, especially apparent during the emergence of novel variants, overwhelms the healthcare systems.

Forecasting the severity of upcoming COVID-19 waves is critical in formulating public health preparedness plans. Knowledge of the population level immunity against SARS-CoV-2 can help to predict the impact of COVID-19 on the healthcare system. COVID-19 vaccination or SARS-CoV-2 infection induce antibody response against SARS-CoV-2^4,5^. A population with high vaccination uptake rate or incidence of infection theoretically have a high level of population immunity. However, several factors can affect the humoral immunity elicited by vaccination or infection^6^. First, some COVID-19 patients or vaccine recipients do not develop sufficient protective antibody responses. Lower neutralizing antibody (NAb) titers are found in patients with asymptomatic or mild COVID-19, immunocompromised patients and older adults^7–13^. Second, the NAb titers elicited by inactivated whole virion or adenovirus-vectored vaccines are lower than those elicited by mRNA vaccines^14–16^. Third, NAb titers decline within a few months after infection or vaccination^17–22^. Fourth, NAb elicited from prior infection or vaccination may not be effective against new variants with mutations in the spike protein, as in the case of the Omicron variant^16,22^.

In view of the shortcomings of using history of vaccination or infection to predict population immunity, we need a more reliable method to determine population level immunity. NAb titers showed a good correlation with vaccine effectiveness against symptomatic infection^14,23^. In a clinical trial of the mRNA-1273 vaccine, the estimated vaccine efficacy decreased from 96% for a NAb titer of 1000 IU/ml to 78% for a NAb titer of 10 IU/ml^24^. Furthermore, individuals positive for anti-spike antibody, which correlates with NAb status, are less likely to have COVID-19 reinfection to cognate variants^25^.

Under zero-case strategy, Hong Kong had a very low incidence of COVID-19, with a cumulative count of just 12,655 cases (0.17% of the population) as of December 2021. There have been four COVID-19 waves in Hong Kong between 2020 and 2021^26–28^. The first wave occurred from January to early March 2020, with the majority being imported cases from mainland China. The second wave occurred from mid-March to May 2020, and consisted of imported cases returning from places outside Asia and the associated local cases. The third and fourth waves occurred from July to August 2020, and from November 2020 to April 2021, respectively. Both the third and fourth waves consisted mainly of locally acquired cases. Local transmission of SARS-CoV-2 was almost absent from May to December 2021.

The COVID-19 vaccination program was launched in Hong Kong in February 2021. Our previous serosurveillance studies showed that <1% of sera collected from the Hong Kong population before March 2021 tested positive for SARS-CoV-2 IgG against the nucleoprotein, but the seropositive rate started to increase from April 2021^29,30^. In the current study, we first screened 1800 serum specimens collected between January and December 2021 with a surrogate virus neutralization test (sVNT), which correlate well with conventional live virus neutralization test (cVNT) against the ancestral virus^22,31^. For sera tested positive by sVNT, we assessed the NAb against the ancestral virus and Omicron sublineages BA.1 and BA.2 using a cVNT. We interpreted our serosurveillance findings with the epidemiological and genomic information of the fifth wave of COVID-19 in Hong Kong, which exceeded 1 million cases by March 18, 2022^32,33^.

## Results

### Sampled population

We retrieved a total of 1800 archived serum specimens collected between January and December 2021 from Queen Mary Hospital, with 300 serum specimens collected every two months. We retrieved approximately the same number of serum specimens from each age group from 0–9 years old to ≥80 years old depending on availability (Supplementary Table S1).

### COVID-19 vaccination in Hong Kong

**Fig. 1:**
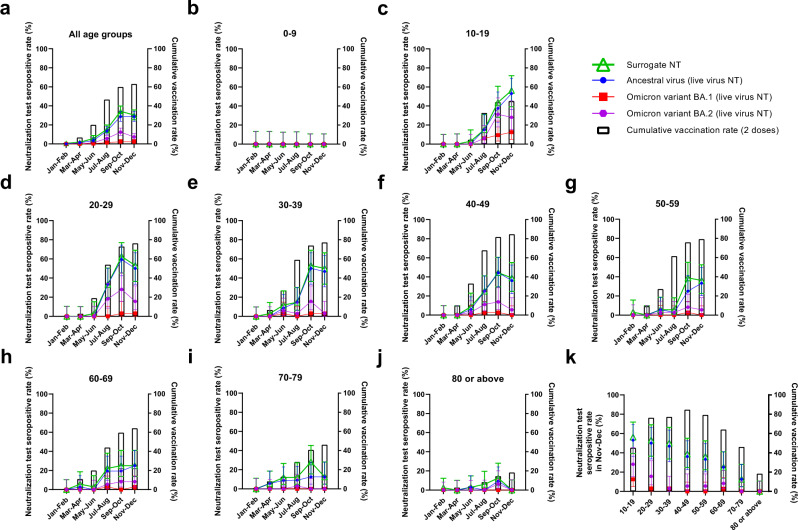
Cumulative two-dose vaccination uptake rates and neutralization test seropositive rates for ancestral virus and Omicron variant sublineages in 2021. **a**–**j** Cumulative two-dose vaccination rates and neutralization test (NT) seropositive rates of different age groups between January and December 2021. **k** Two-dose cumulative vaccination uptake rates and neutralization test seropositive rates in November/December 2021. **a**–**k** Cumulative vaccination rate represents the percentage of the population that have received 2 doses of COVID-19 vaccines at the end of each time period^34^. Neutralization test seropositive rates represent the percentage of sera tested positive in each time period. All serum specimens were tested with the surrogate virus neutralization test (sVNT) (iFlash-2019-nCoV neutralization antibody assay), which measures antibodies that block the interaction between ancestral virus receptor binding domain (RBD) and human angiotensin-converting enzyme 2 (ACE2). Serum specimens tested positive in the sVNT were then tested for NAb against the ancestral virus, Omicron sublineage BA.1 and Omicron sublineage BA.2 using a conventional live virus neutralization test (cVNT). A serum specimen is considered to be seropositive in the sVNT if ≥15 AU/ml, and in the cVNT if the NAb titer is ≥10. The number of serum specimens tested in each period is shown in Supplementary Table S1. For neutralization test seropositive rates, the symbols indicate the mean, and the error bars indicate 95% confidence interval. Source data are provided as a Source Data file.

Hong Kong’s COVID-19 vaccination program was launched in February 2021^34^ (Table 1). BNT162b2 and CoronaVac are the only vaccines available. Overall, 63.0% (4,662,004/7,394,700) of the population in Hong Kong has received the second dose of COVID-19 vaccines by December 31, 2021 (Fig. 1a). The two-dose vaccination rate was highest for the 40–49 year-old age group (84.7%; 967,935/1,142,500), and lowest for the ≥80 year-old age group (18.4%; 73,772/401,800) (Fig. 1b–j). The highest number of second-dose vaccination occurred in July/August, with much fewer people receiving the second dose in September/October and November/December (Supplementary Fig. S1).

**Table 1:** Timeline of COVID-19 vaccination program in Hong Kong.

Date	Event
26th February 2021	Administration of CoronaVac (Sinovac) vaccine officially started for adults aged 18 years or above
10th March 2021	Administration of BNT162b2 (Pfizer-BioNTech) vaccine officially started for adults aged 18 years or above
14th June 2021	Administration of BNT162b2 (Pfizer-BioNTech) vaccine started for adolescents aged 12–17 years old
2nd December 2021	Administration of CoronaVac (Sinovac) vaccine started for adolescents aged 12–17 years old

**Fig. 2:**
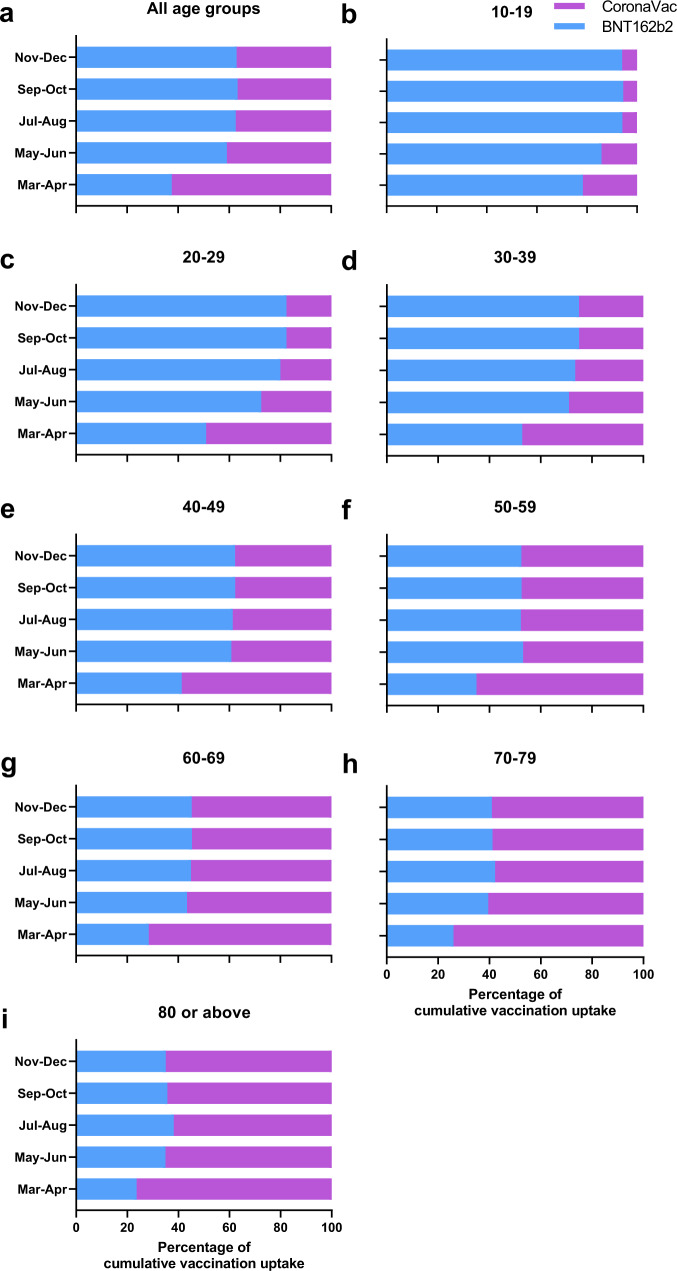
Percentage of individuals receiving BNT162b2 or CoronaVac in Hong Kong in 2021. **a**–**i** Percentage of individuals receiving BNT162b2 or CoronaVac for different age groups. The data were extracted from the website of the Food and Health Bureau of Hong Kong^34^. Source data are provided as a Source Data file.

Overall, 63.0% (2,936,358 / 4,662,004) and 37.0% (1,725,646/ 4,662,004) received BNT162b2 and CoronaVac, respectively. However, the proportion of individuals receiving BNT162b2 was higher among the younger age groups than older age groups. For the 10–19 year-old age group, 94.0% (241,813 / 257,242) received BNT162b2, while there was a decreasing trend for older age groups (Fig. 2a–i). For the 80 years or above age group, only 35.1% (25,869 / 73,772) received the BNT162b2 vaccine.

### Surrogate virus neutralization test

We first screened all 1800 serum specimens with a sVNT (iFlash-2019-nCoV NAb assay, YHLO). The sVNT NAb result between January and April 2021 was reported previously^29^. Overall, there was an increase in the proportion of individuals tested positive in the sVNT in March/April after the launch of the COVID-19 vaccination program. The sVNT seropositive rate increased from 1.7% (5/300) (95% confidence interval [CI]: 0.7–3.8%) in March/April to a peak of 34.3% (103/300) (95% CI: 29.2–39.9%) in September/October, with a slight decline to 30.3% (91/300) (95% CI: 25.4–35.8%) in November/December 2021 (Fig. 1a). The geometric mean sVNT levels also peaked in September/October, and declined in all age groups in November/December except for the 10–19 year-old age group (Supplementary Fig. S2).

**Fig. 3:**
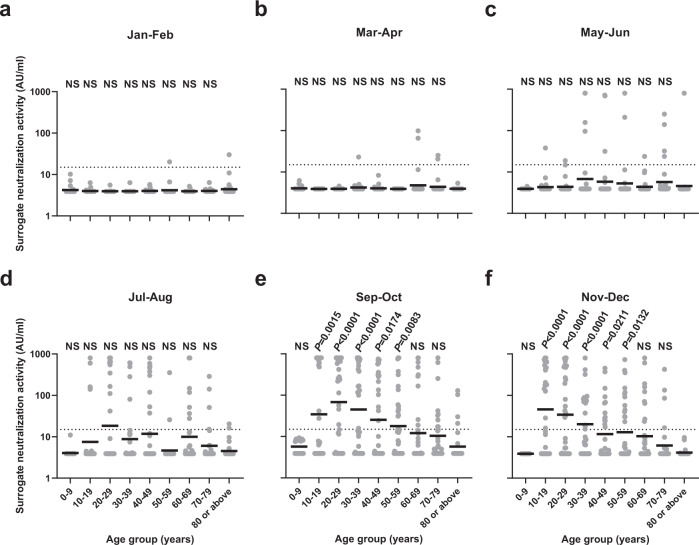
Comparison of surrogate neutralization activity among different age groups. **a**–**f** Surrogate neutralization activity in different time periods. The horizontal bar indicates the geometric mean level. Dotted horizontal lines represent the manufacturer’s seropositivity cutoff level (15 AU/ml). The geometric mean antibody titer for each age group was compared to the ≥80 years age group using the one way ANOVA with Dunn’s multiple comparisons test. The number of serum specimens tested in each period is shown in Supplementary Table S1. Source data are provided as a Source Data file. NS, not significant.

Subgroup analysis of different age groups revealed stark differences in the positive rate in the sVNT among different age groups (Fig. 1b–j). For sera collected from the 0–9 year-old individuals, who were not eligible for COVID-19 vaccines in 2021, all specimens tested negative in the sVNT (Fig. 1b). For the 10–19 year-old age group, the seropositive rate rose sharply after July 2021, which coincided with the commencement of COVID-19 vaccination program for adolescents aged 12–17 years old on 14th June 2021 (Fig. 1c)^35^. In November/December, the 10–19 year-old age group had the highest sVNT seropositive rate, and there was a statistically significant trend towards lower seropositive rate for older age groups (age trend by Cochran-Armitage trend test; *P* < 0.0001 for age groups between 30–39 and ≥80 years) (Fig. 1k). Notably, none of the sera from the ≥80 year-old age group tested positive in the sVNT in November/December. The 10–19 year-old age group also had the highest sVNT activity (Fig. 3), and there was a statistically significant trend towards lower sVNT activity for older adults (*P* = 0.0014); excluding the 0–9 year-old age group (Fig. 3f).

### Conventional live virus neutralization tests

The commercial sVNT assay in this study was designed based on the ancestral virus. Our previous study showed that this assay has a high concordance with the result of cVNT NAb against the ancestral virus, but poor correlation with cVNT NAb against the Omicron sublineage BA.1 due to immune escape^22^. Hence, for specimens tested positive in the sVNT, we used a cVNT to determine the NAb activity against the Omicron sublineages BA.1 and BA.2 and the ancestral virus. Overall, 86.0% (227/264) of sVNT positive specimens had detectable NAb against the ancestral virus (Fig. 1a). In contrast, only 7.2% (19/264) and 30.3% (80/264) had detectable NAb against BA.1 and BA.2, respectively. Live virus NAb against BA.1 was only found in sera from individuals between 10 and 69 years old, whereas live virus NAb activity against BA.2 could be found in sera from all age groups except for the 0–9 year-old age group (Fig. 1b–j). Assuming that all sVNT negative sera did not have detectable NAb against BA.1 or BA.2 (since our previous study showed that sVNT has 100% sensitivity for NAb against the Omicron variant^22^), the proportion of individuals with detectable NAb against BA.1 and BA.2 in November/December 2021 were 2.3% (7/300) and 7.3% (22/300), respectively.

The absence of seropositive sera in the 0–9 year-old age group, the temporal association of seropositive rate and two-dose vaccination rate, and the low incidence of locally acquired COVID-19 cases after April 2021, suggested that NAb were elicited by COVID-19 vaccination in most of the seropositive individuals in our population. While the two-dose vaccination rate and sVNT positive rates were very similar in the younger age groups, there was a greater discrepancy between the vaccination and sVNT seropositive rates in the older age groups (Fig. 1). In November/December, the sVNT seropositive rate was <50% for the 40–49 year-old or older age groups. The greater discrepancy between vaccination and sVNT seropositive rates may be related to the lower proportion of individuals in the older age group receiving BNT162b2 (Fig. 2), as the seropositive rate and antibody titer has been shown to be higher for BNT162b2 than CoronaVac^16,36–38^.

### The fifth wave of COVID-19 in Hong Kong

**Fig. 4:**
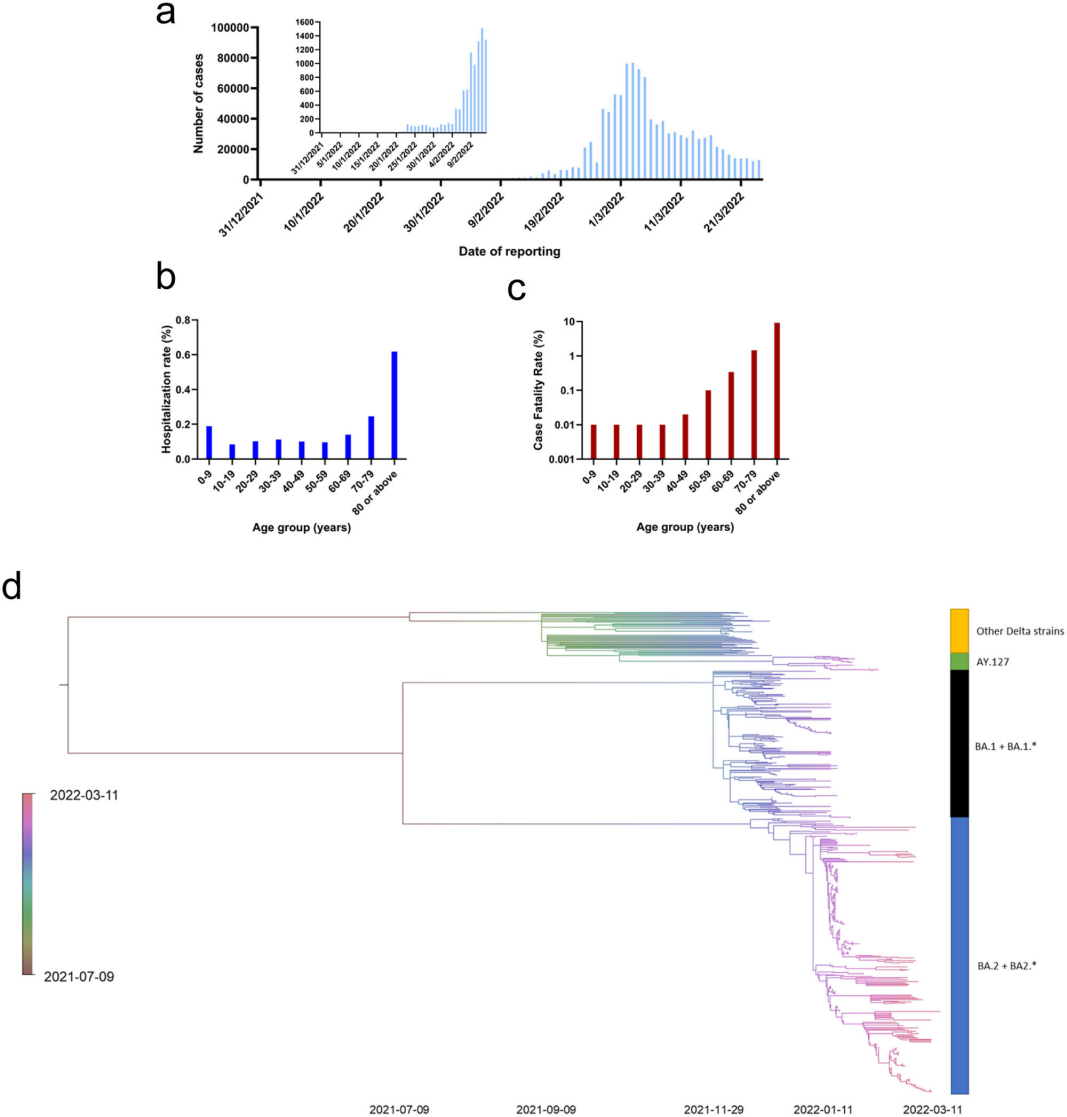
Epidemiology and genomic analysis of the fifth wave of COVID-19 in Hong Kong. **a** Epidemic curve showing the incidence of COVID-19 cases between December 31, 2021 and March 24, 2022. **b** Age specific incidence rate of COVID-19 related hospitalization in Hong Kong as of February 24, 2022. **c** Age specific case-fatality rate of COVID-19 as of March 24, 2022^44^. **d** Time-resolved phylogenetic tree of 545 viral genomes from Dec 1, 2021 to March 11 2022. BA.1.* includes BA.1.1, BA.1.1.1, BA.1.1.10, BA.1.1.13, BA.1.1.14, BA.1.1.16, BA.1.1.18, BA.1.1.2, BA.1.14, BA.1.15, BA.1.15.1, BA.1.17, BA.1.17.2 and BA.1.18. BA.2.* includes BA.2.2, BA.2.3, and BA.2.10. Details of the sequences used are shown in Supplementary Data 1. Source data are provided as a Source Data file.

In Hong Kong, the fourth wave occurred between November 2020 and April 2021. Only five cases were reported between May 12 and October 8, 2021, and no locally acquired cases were reported between October 9 and Dec 30, 2021. The first local case of the fifth wave was reported on Dec 31, 2021. There was limited local transmission in January 2022 due to several Omicron variant-related clusters which could be traced to imported cases (*n* = 836), including a BA.1 outbreak in a restaurant and a large Omicron BA.2 outbreak in a housing estate which we reported previously^39,40^ (Fig. 4a). There was also a cluster of pet-store-related Delta variant AY.127^41^. Since early February, 2022, there was an exponential increase in cases from <200 cases per day before February 4 to a peak of 76,991 cases (56,827 confirmed by nucleic acid test and 20,164 confirmed by self-collected rapid antigen test) on March 3, 2022^42,43^. As of March 24, 2022, there were 1,087,610 cases reported during the fifth wave. The age specific rate for COVID-19 hospitalization was highest among older adults ≥80 years: 0.62% [2,485/401,800]; 70–79 years old: 0.25% [1,381/560,500] and children (0–9 years old: 0.19%) [1,013/538,500] (Fig. 4b). For the age groups from 10–59, the hospitalization rate was between 0.08–0.12%. The overall case-fatality rate was 0.60%, but the case-fatality rates were 15.3-fold and 2.4-fold higher for the ≥80 (9.19%) and 70–79 (1.46%) year-old age groups, respectively^44^ (Fig. 4c).

### Genomic epidemiology of the fifth wave from January to March 2022

We have previously reported our viral genome sequencing results for specimens collected up to February 2, 2022, including those from an Omicron BA.1 restaurant outbreak^40^, an Omicron BA.2 housing estate outbreak^39^, and a Delta AY.127 outbreak related to pet stores^41^. Here, we have performed additional sequencing for specimens collected in Hong Kong up to March 11, 2022 (Supplementary Data 1).

In total, 545 specimens collected between December 1, 2021 and March 11, 2022, including 383 specimens collected during the fifth wave (on or after December 31, 2021), were analyzed in a time-resolved phylogenetic tree. During the fifth wave, 80.2% (307/383) of viral genome sequences belonged to Omicron sublineage BA.2 or related sublineages (BA.2.2, BA.2.3 or BA.2.10), 15.4% (59/383) belonged to Omicron sublineage BA.1 or related sublineages (BA.1.*), and 4.4% (17/383) belonged to Delta variant AY.127 (Fig. 4d). Notably, all 127 viral genomes from specimens collected on or after February 12, 2022, belonged to the sublineages BA.2, BA.2.2, or BA.2.10 (Supplementary Data 1). The majority of (98.4%; 125/127) belonged to BA.2.2, which is characterized by the mutations C12525T (ORF1a T4087I)^45^.

## Discussion

Serosurveillance is an effective tool to reveal the hidden burden of infection, especially at the beginning of the COVID-19 pandemic^30^. In addition, serosurveillance using NAb assays is useful in assessing the level of protective immunity in a population. Our study found that although the cumulative two-dose vaccination uptake rate exceeded 60% in Hong Kong by November 2021, only 30% of our study population had a positive sVNT in November/December 2021; and only 2.3% and 7.3% had detectable NAb against the Omicron sublineages BA.1 and BA.2, respectively. In particular, older adults had the lowest levels of protective immunity. In November/December 2021, none of the ≥80 year-old age group and only 12.5% of the 70–79 year-old age group had a positive sVNT, while none of the individuals ≥70 years old had detectable NAb against BA.1 or BA.2. The lack of protective immunity against the Omicron BA.2 sublineage, especially among older adults, likely contributed to the severe fifth wave in Hong Kong which exponentially increased in February 2022 and overwhelmed the healthcare system in Hong Kong^46^.

Vaccination is associated with a much lower risk of death^33^. Unfortunately, the vaccination rate was particularly low among older adults aged 80 years or above. In a study conducted in mainland China, the two main reasons for vaccine hesitancy among adults aged 60 years or above were concern for vaccine safety and low infection risk^47^. In another study, Lau et al. showed that the presence of health conditions was one of the major factors associated with vaccine hesitancy or resistance among adults in Hong Kong^48^. Many older adults with comorbidities believe that vaccination may exacerbate their underlying diseases. In Hong Kong, the low incidence of COVID-19 in Hong Kong before the fifth wave have led to a low perceived infection risk.

The sVNT seropositive rate was very similar to the two-dose cumulative vaccination rate among the 10–19 and 20–29 year-old age groups. However, we observed a greater discrepancy between two-dose vaccination rate and sVNT seropositive rate for older adults, which may be related to the weaker immune response in this age group. Previous studies showed that older adults have poorer antibody response after vaccination compared to younger adults. Saure et al. showed that the IgG seropositive rate after vaccination was lower among adults aged ≥60 years than those aged 18–39 years^17^. Wei et al. showed that seropositivity after vaccination reduced faster for vaccine recipients aged over 75 years^12^. Another possible reason for the low seropositive rate among older adults is the vaccine preference among these age groups. Over 50% of adults aged ≥60 years have chosen CoronaVac. Head-to-head comparison showed that CoronaVac recipients had lower seropositive rates than BNT162b2 recipients after 2 doses^13,16^. Furthermore, seropositive rate declined more rapidly for the CoronaVac than BNT162b2 recipients^17^. Our results support the World Health Organization’s recommendation that older adults should receive three doses of CoronaVac^49^.

The incidence of COVID-19 was very low in Hong Kong as a result of elimination strategy. Hence, the NAb detected by sVNT or cVNT for most individuals is likely elicited by vaccination rather than by infection. For the 0–9 year-old age group, none of the serum specimens tested positive in the sVNT. This is expected as the vaccination program for the 0–9 year-old age group was not yet started in 2021 and the prevalence of infection is likely to be very low as the number of laboratory-confirmed cases was only 0.1% (547/538500) of the population.

There was a decrease in seropositive rates in November/December. This coincided with a low vaccination update rate since September (Supplementary Fig. S1), suggesting that there is waning of humoral immunity. Our previous study showed that for natural infection, waning of NAb level occurs most rapidly in the first 4 months^22^. For vaccination, there is also evidence of waning immunity within the first few months^50^.

The spike protein of Omicron sublineage BA.2, especially the RBD, differs from that of BA.1. There were conflicting data regarding the difference in NAb titer against BA.1 and BA.2 for post-vaccine sera. In our previous study using serum from vaccine recipients or COVID-19 patients^51^ and in this current study, we showed that BA.2 NAb titers were generally higher than those of BA.1. Iketani et al. found that the NAb titers were similar between BA.1 and BA.2^52^, while Yamashobe et al. and Yu et al. showed a slightly lower NAb titer against BA.2^53,54^. The difference may be related to the assays used. Both Yamashobe and Yu et al used pseudovirus assay, while our studies and those of Iketani et al used cVNT.

sVNT is a convenient assay which can be conducted in clinical or research laboratories without biosafety-level 3 facilities. sVNT is a competitive assay which measures antibodies that prevent the binding between SARS-CoV-2 RBD and the human receptor ACE2. Our previous study showed that sVNT has a sensitivity and specificity of 97.9% and 94.9%, using cVNT as the gold standard^55^. Our current study also showed a good correlation between sVNT and live virus NAb test against the ancestral virus for sVNT seropositive individuals. The sVNT can be easily modified to measure antibodies against the RBD of Omicron sublineages, and this would allow a more convenient assay for serosurveillance in the future.

There are several limitations in this study. First, as we are using anonymized serum specimens, we do not have clinical information or vaccination history from these individuals. Therefore, we are not able to perform further analysis to study factors that affect NAb response. In a population study conducted in UK, Wei et al showed that ethnicity, sex, social deprivation, and long term health conditions were associated with seropositivity after vaccination^12^. Second, this study included hospital patients instead of the “general population”. Therefore, our data may not be representative of people without comorbidities. However, those with underlying diseases would have a much greater risk of having severe disease, and therefore our estimate would be better in predicting the burden to the healthcare systems. Third, there were fewer specimens in the pediatric age groups as the volume of archived serum specimens were not sufficient for testing. Fourth, we did not test T cell immunity. Future studies are required to understand the relationship between population T cell immunity and susceptibility to infection.

Our study highlights the value of NAb surveillance in assessing population immunity. We demonstrated that the level of protective immunity, especially against antigenically distinct variants, may not be reliably predicted by vaccination history. The low level of protective immunity among older adults was associated with an exceptionally high case-fatality rate. Vaccine studies should put more focus on the older adults. Improving the immunogenicity among older adults should be a priority in vaccine research.

## Methods

### Patients

Anonymised archived serum samples from the clinical biochemistry laboratory of Queen Mary Hospital in Hong Kong were used for serological surveillance as we described previously^29,30^. The archived specimens encompassed all age groups from 0–9 years old to ≥80 years old. A total of 300 serum specimens were randomly selected and tested in each two-month period from January/February to November/December 2021, with an approximately equal number of archived specimens in each 10-year age group depending on availability (Supplementary Table S1). Specimens were excluded if there was an insufficient volume of serum. Results of the surrogate NAb and live virus NAb against the ancestral virus for serum specimens collected between January and April 2021 was reported in our previous publication^29^, but the result of NAb against the Omicron sublineages BA.1 or BA.2 has not been reported before. For viral genome sequencing, 382 sequences were reported in our previous publications^39–41^. In the current study, we have performed genome sequencing for 163 randomly selected archived respiratory specimens collected from patients admitted to Queen Mary Hospital. This study was approved by the Institutional Review Board of the University of Hong Kong/Hospital Authority Hong Kong West Cluster (UW 21-313 and UW 18–141). Written informed consent was waived since archived anonymized specimens were used.

### Surrogate neutralization test and live virus neutralization test

sVNT test was performed using iFlash-2019-nCoV neutralization antibody assay according to manufacturer’s instructions (Shenzhen YHLO Biotech Co. Ltd., Shenzhen, China)^56^. The manufacturer’s cutoff for seropositivity is 15 AU/ml, while the maximum measurable result is 800 AU/ml.

cVNT was performed on VeroE6/TMPRSS2 cells (JCRB cell bank of Okayama University; Cat#JCRB1819). The cVNT NAb titer was determined by an end-point dilution and cytopathic effect as we described previously^16^. Briefly, serum specimens were heat inactivated at 56 °C for 30 min and were serially diluted in 2-folds with MEM containing 1% fetal calf serum (FBS) (Gibco). Duplicates of each diluted serum were mixed with 100 TCID_50_ of an ancestral virus (GISAID accession number: EPI_ISL_434571), an Omicron sublineage BA.1 virus (GISAID accession number: EPI_ISL_7138045)^51^, or an Omicron sublineage BA.2 virus (GISAID accession number: EPI_ISL_9845731)^51^ at 37 °C for 1 h. After incubation, 100 μL of the serum-virus mixture was then added to VeroE6/TMPRSS2 cells that were seeded in 96-well plates 24 h before infection. The cells were incubated with the mixture at 37 °C. After incubation for 3 days, cytopathic effect was examined. The cVNT was determined as the highest dilution with 50% inhibition of cytopathic effect. A cVNT of ≥10 was considered positive. For statistical analysis, a value of 5 was assigned if the cVNT is <10.

### Whole genome sequencing and genome data analysis

Whole genome sequencing was performed using the Oxford Nanopore MinION device (Oxford Nanopore Technologies) as we described previously^16^. For the determination of viral lineage, nanopore sequencing was performed following the Nanopore protocol - PCR tiling of COVID-19 (Version: PTC_9096_v109_revH_06Feb2020) according to the manufacturer’s instructions with minor modifications (Oxford Nanopore Technologies) as we described previously^26,27^. Briefly, extracted RNA was first reverse transcribed to cDNA using SuperScript^TM^ IV reverse transcriptase (ThermoFisher Scientific, Waltham, MA, USA). PCR amplification was then performed using the hCoV-2019/nCoV-2019 Version 3 Amplicon Set (Integrated DNA Technologies, Coralville, IA, USA) with the Q5® Hot Start High-Fidelity 2X Master Mix kit (New England Biolabs, Ipswich, Massachusetts, United States) according to the Nanopore protocol. PCR products were purified using 1x AMPure XP beads (Beckman Coulter, Brea, CA, USA) and quantified using Qubit dsDNA HS Assay Kit (Thermo Fisher Scientific, Waltham, Massachusetts, United States). The purified DNA was then normalized for end-prep and native barcode ligation reactions according to the PCR tiling of COVID-19 virus protocol with Native Barcoding Expansion 96 (EXP-NBD196, Oxford Nanopore Technologies). Barcoded libraries were then pooled, purified with 0.4x AMPure XP beads and then quantified using Qubit dsDNA HS Assay Kit. Purified pooled libraries were ligated to sequencing adapters and sequenced with the Oxford Nanopore MinION device using R9.4.1 flow cells for 24–48 h.

For bioinformatics analysis, the recommended ARTIC bioinformatics workflow (version 1.2.1) was used with minor modifications applied as described previously^26,27^. The modifications include reducing the minimum length at the guppyplex step to 350 to allow potential small deletions to be detected and increasing the “–normalise” value to 999999 to incorporate all the sequenced reads and the high accurate mode was used for basecalling with an increased QC passing score from 7 to 10. The sequence NC_045512.2 obtained from NCBI was used as the reference and the alignment files produced by Medaka were inspected using Integrative Genomics Viewer (IGV) (2.8.0) to verify the mutations called by the ARTIC pipeline. SARS-CoV-2 lineage was assigned using the PANGOLIN software suite (v4.0.6; accessed on May 6, 2022)^57^. All sequences were deposited onto the NCBI and GISAID database (Supplementary Data 1).

### Time-resolved phylogenetic tree

Time-resolved phylogenetic tree was constructed using TreeTime program (version 0.9.0-b.2) as we described previously^27^. In addition to the described homoplastic position masking, we also removed the highly diverged sequences suggested by the TreeTime program. Sequences collected from December 1, 2021 to March 11, 2022 were aligned using MAFFT aligner (v7.310). The maximum-likelihood whole genome phylogenetic tree construction and phylodynamic analysis were performed using IQ-TREE (multicore version 2.2.0 COVID-edition) and TreeTime. For the construction of the phylogenetic tree, 100 standard nonparametric bootstrap replicates were used, and the option -czb was used to mask the unrelated substructure of the tree with branch length representing mutation count of less than 1. The resulting tree, sample collection date and the aligned sequences were used as an input for the TreeTime pipeline to create the phylodynamic tree. The outlier sequences (flagged by the TreeTime program) were removed when visualizing the tree.

### Data for two-dose vaccination rate and COVID-19 outbreak in HK

The data for the two-dose vaccination uptake rate in Hong Kong was obtained from the Hong Kong government webpage^34^. The data of the Hong Kong population was obtained from Census and Statistics Department^58^.

### Statistical analysis

Statistical analysis was performed using SPSS 26.0 (IBM SPSS Statistics) and GraphPad PRISM 9.1.1 (GraphPad Software, San Diego CA, USA). The Cochran-Armitage trend test was used to assess the age trend in the seropositive rate of neutralization test. The simple linear regression was used to assess the age trend in the sVNT titer. The geometric mean antibody titer for each age group was compared to the ≥80 years age group using the one way ANOVA with Dunn’s multiple comparisons test. For the purpose of statistical analysis, an MN titer of <10 was considered as 5. A *P* value of <0.05 was considered statistically significant.

### Reporting summary

Further information on research design is available in the Nature Research Reporting Summary linked to this article.

## Supplementary information

Supplementary Information

Description of Additional Supplementary Files

Supplementary Data 1

Reporting Summary

## Data Availability

The source data on sVNT and cVNT titers in this study have been deposited into GitHub (https://github.com/SMUAbdullah/paper-Omicron-BA.2-outbreak-Hong-Kong). The genome sequences have been deposited into the NCBI GenBank and GISAID database (Supplementary Data 1). Data of the vaccination update rate in Hong Kong was obtained from the Food and Health Bureau of the HKSAR government website https://data.gov.hk/en-data/dataset/hk-fhb-fhbcovid19-vaccination-rates-over-time-by-age. The data of the Hong Kong population was obtained from the Census and Statistics Department of the HKSAR government website https://www.censtatd.gov.hk/en/web_table.html?id=1A.
