# Supplementary material for: Contribution of low population immunity to the severe Omicron BA.2 outbreak in Hong Kong
Source: Nat Commun. 2022 Jun 24;13:3618. doi: 10.1038/s41467-022-31395-0 (PMC9232516; doi:10.1038/s41467-022-31395-0)
Supplement: Supplementary file 1 — Supplementary Information [file 41467_2022_31395_MOESM1_ESM.pdf]

Supplementary Information

Contribution of low population immunity to the severe Omicron BA.2 outbreak in Hong Kong

Authors: Lin-Lei Chen[1*], Syed Muhammad Umer Abdullah[1*], Wan-Mui Chan[1], Brian Pui-Chun Chan[1], Jonathan Daniel Ip[1], Allen Wing-Ho Chu[1], Lu Lu[1], Xiaojuan Zhang[1], Yan Zhao[1], Vivien Wai-Man Chuang[2], Albert Ka-Wing Au[3], Vincent Chi-Chung Cheng[1,4,5], Siddarth Sridhar[1,4,7], Kwok-Yung Yuen[1,4,6,7], Ivan Fan-Ngai Hung[8], Kwok-Hung Chan[1,6], Kelvin Kai-Wang To[1,4,6,7#]

Author affiliations:
[1] State Key Laboratory for Emerging Infectious Diseases, Carol Yu Centre for Infection, Department of Microbiology, School of Clinical Medicine, Li Ka Shing Faculty of Medicine, Pokfulam, The University of Hong Kong, Hong Kong Special Administrative Region, China.
[2] Quality and Safety Division, Hospital Authority, Hong Kong Special Administrative Region, China
[3] Centre for Health Protection, Department of Health, Hong Kong Special Administrative Region, China
[4] Department of Microbiology, Queen Mary Hospital, Pokfulam, Hong Kong Special Administrative Region, China.
[5] Infection Control Team, Queen Mary Hospital, Hong Kong West Cluster, Hong Kong Special Administrative Region, China.
[6] Department of Clinical Microbiology and Infection Control, The University of Hong Kong-Shenzhen Hospital, Shenzhen, China
[7] Centre for Virology, Vaccinology and Therapeutics, Hong Kong Science and Technology Park, Hong Kong Special Administrative Region, China.
[8] Department of Medicine, School of Clinical Medicine, Li Ka Shing Faculty of Medicine, Pokfulam, The University of Hong Kong, Hong Kong Special Administrative Region, China.

* Equal contribution
[#] Corresponding author: Kelvin Kai-Wang To. Email: kelvinto@hku.hk

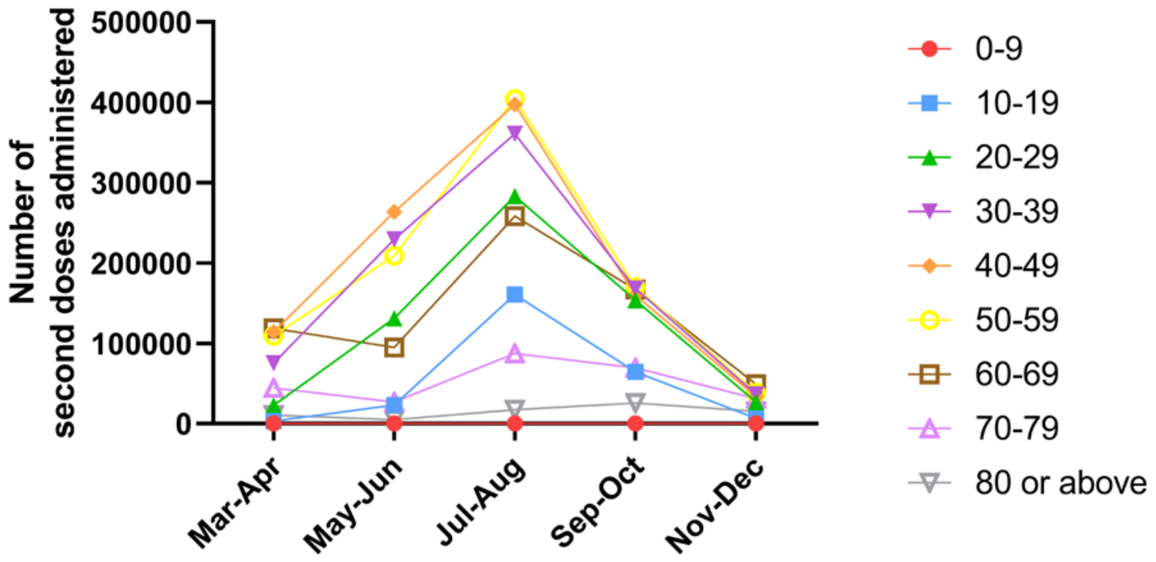

Supplementary Figure S1. **Number of individuals with second dose of COVID-19 vaccine administered in each time period.** Data were obtained from the website of the Food and Health Bureau of Hong Kong [32]. Source data are provided as a Source Data file.

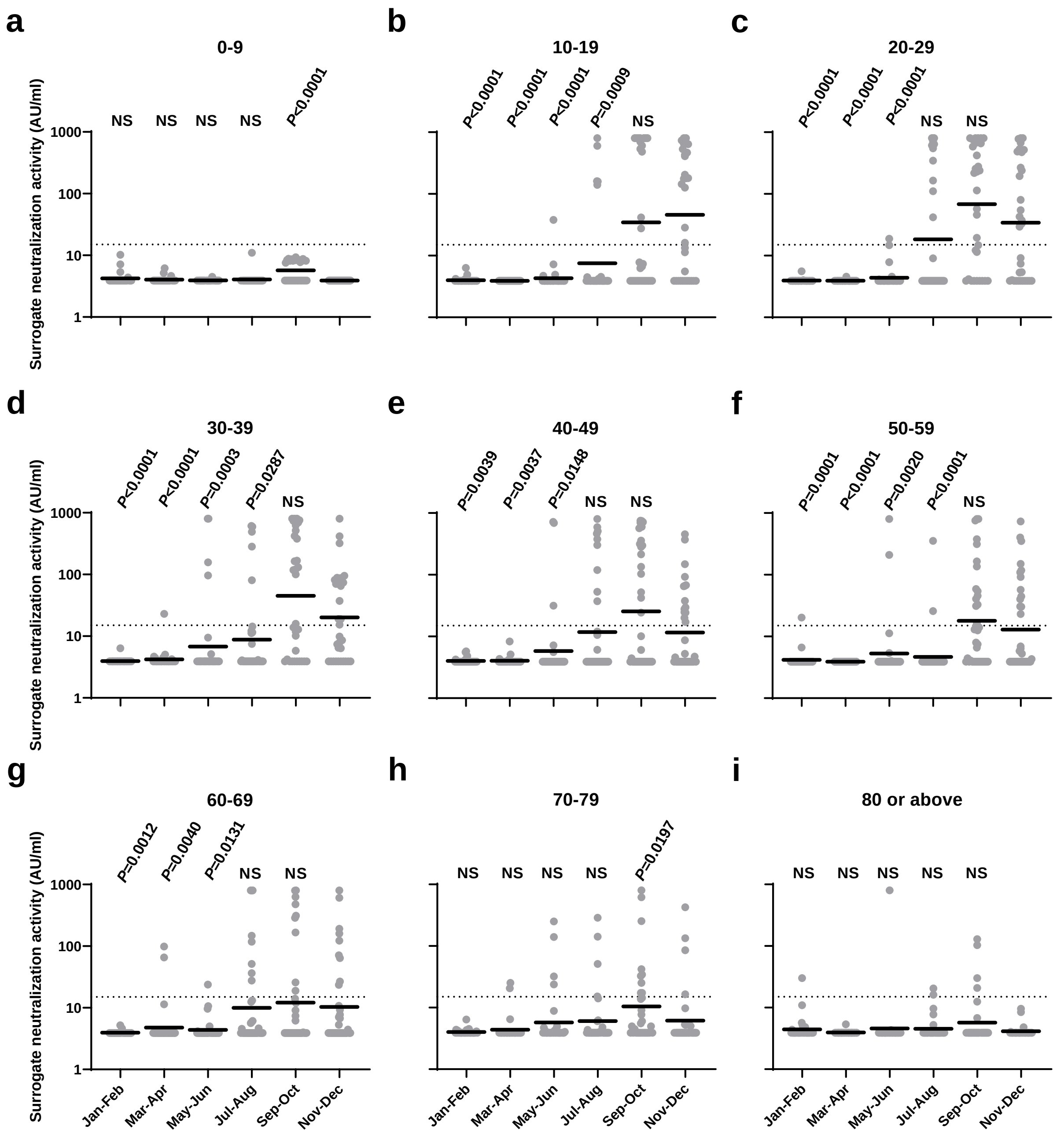

Supplementary Figure S2. The levels of neutralizing antibody in the surrogate virus neutralization test. (a-i) The levels of neutralizing antibody in different age groups. The geometric mean level is shown in the horizontal bar. Dotted horizontal lines represent the manufacturer's seropositivity cutoff level (15 AU/ml). The geometric mean antibody titer

for each age group was compared to the ≥80 years age group using the one way ANOVA with Dunn's multiple comparisons test. The number of serum specimens tested in each period is shown in Supplementary Table S1. Source data are provided as a Source Data file. Abbreviations: ns, not significant

Supplementary Table S1. Number of serum specimens tested by surrogate virus neutralization test.

Age group	Jan/Feb 2021	Mar/Apr 2021	May/Jun 2021	July/Aug 2021	Sep/Oct 2021	Nov/Dec 2021	Total
0-9	25	25	27	26	32	32	167
10-19	34	32	34	33	32	32	197
20-29	33	34	33	33	32	32	197
30-39	35	35	34	34	32	32	202
40-49	35	35	34	36	36	36	212
50-59	32	35	35	36	36	36	210
60-69	33	35	35	36	36	36	211
70-79	31	35	34	33	32	32	197
>=80	42	34	34	33	32	32	207
Total	300	300	300	300	300	300	1800
