# Supplementary material for: Contribution of low population immunity to the severe Omicron BA.2 outbreak in Hong Kong
Source: Nat Commun. 2022 Jun 24;13:3618. doi: 10.1038/s41467-022-31395-0 (PMC9232516; doi:10.1038/s41467-022-31395-0)
Supplement: Supplementary file 2 — Description of Additional Supplementary Files [file 41467_2022_31395_MOESM2_ESM.pdf]

Description of Additional Supplementary Files

File Name: Supplementary Data 1
Description: Details of sequences used in the time-resolved phylogenetic tree
