# Supplementary material for: Contribution of low population immunity to the severe Omicron BA.2 outbreak in Hong Kong
Source: Nat Commun. 2022 Jun 24;13:3618. doi: 10.1038/s41467-022-31395-0 (PMC9232516; doi:10.1038/s41467-022-31395-0)
Supplement: Supplementary file 4 — Reporting Summary [file 41467_2022_31395_MOESM4_ESM.pdf]

nature portfolio

Corresponding author(s): Kelvin To

Last updated by author(s): Jun 14, 2022

Reporting Summary

Nature Portfolio wishes to improve the reproducibility of the work that we publish. This form provides structure for consistency and transparency in reporting. For further information on Nature Portfolio policies, see our Editorial Policies and the Editorial Policy Checklist.

Statistics

For all statistical analyses, confirm that the following items are present in the figure legend, table legend, main text, or Methods section.

n/a	Confirmed	
☐	☒	The exact sample size (*n*) for each experimental group/condition, given as a discrete number and unit of measurement
☐	☒	A statement on whether measurements were taken from distinct samples or whether the same sample was measured repeatedly
☐	☒	The statistical test(s) used AND whether they are one- or two-sided<br>*Only common tests should be described solely by name; describe more complex techniques in the Methods section.*
☒	☐	A description of all covariates tested
☒	☐	A description of any assumptions or corrections, such as tests of normality and adjustment for multiple comparisons
☐	☒	A full description of the statistical parameters including central tendency (e.g. means) or other basic estimates (e.g. regression coefficient) AND variation (e.g. standard deviation) or associated estimates of uncertainty (e.g. confidence intervals)
☐	☒	For null hypothesis testing, the test statistic (e.g. *F*, *t*, *r*) with confidence intervals, effect sizes, degrees of freedom and *P* value noted<br>*Give P values as exact values whenever suitable.*
☒	☐	For Bayesian analysis, information on the choice of priors and Markov chain Monte Carlo settings
☒	☐	For hierarchical and complex designs, identification of the appropriate level for tests and full reporting of outcomes
☒	☐	Estimates of effect sizes (e.g. Cohen's *d*, Pearson's *r*), indicating how they were calculated

Our web collection on statistics for biologists contains articles on many of the points above.

Software and code

Policy information about availability of computer code

Data collection	We did not use any software for data collection.
Data analysis	GraphPad PRISM 9.1.1 was used for analysis and plotting the figures. SPSS 26.0 (IBM SPSS Statistics) was used for statistical analysis. Bioinformatics analysis was performed using the ARTIC bioinformatics workflow (1.2.1). Sequences were aligned using MAFFT aligner (v7.310). Alignment files were visualized using Integrative Genomics Viewer (IGV) (2.8.0). Time-resolved phylogenetic tree was constructed using TreeTime program (0.9.0-b.2). SARS-CoV-2 lineage was assigned using the PANGOLIN software suite (v4.0.6; accessed on May 6, 2022)

For manuscripts utilizing custom algorithms or software that are central to the research but not yet described in published literature, software must be made available to editors and reviewers. We strongly encourage code deposition in a community repository (e.g. GitHub). See the Nature Portfolio guidelines for submitting code & software for further information.

Data

Policy information about availability of data

All manuscripts must include a data availability statement. This statement should provide the following information, where applicable:

- Accession codes, unique identifiers, or web links for publicly available datasets
- A description of any restrictions on data availability
- For clinical datasets or third party data, please ensure that the statement adheres to our policy

The source data on sVNT and cVNT titers in this study have been deposited into GitHub (https://github.com/SMUAbdullah/paper-Omicron-BA.2-outbreak-Hong-Kong). The genome sequences have been deposited into the NCBI GenBank and GISAID database (Supplementary Data 1). Data of the vaccination update rate in Hong Kong was obtained from the Food and Health Bureau of the HKSAR government website https://data.gov.hk/en-data/dataset/hk-fhb-fhbcovid19-vaccination-

rates-over-time-by-age. The data of the Hong Kong population was obtained from the Census and Statistics Department of the HKSAR government website https://www.censtatd.gov.hk/en/web_table.html?id=1A.

Field-specific reporting

Please select the one below that is the best fit for your research. If you are not sure, read the appropriate sections before making your selection.

☒ Life sciences ☐ Behavioural & social sciences ☐ Ecological, evolutionary & environmental sciences

For a reference copy of the document with all sections, see nature.com/documents/nr-reporting-summary-flat.pdf

Life sciences study design

All studies must disclose on these points even when the disclosure is negative.

Sample size	This study included a total of 1800 serum specimens and 565 respiratory specimens. For serum specimens, the sample size was chosen based on specimen availability and to ensure approximately equal number of specimens for each age group. For respiratory specimens, the sample size was based on the availability of specimens.
Data exclusions	Serum specimens were excluded if the volume is insufficient for antibody testing
Replication	The neutralizing antibody titers were performed in duplicates.
Randomization	Anonymised serum samples from the clinical biochemistry laboratory were randomly selected for neutralization assay. As this is an observational study, there was no group allocation.
Blinding	Blinding is not relevant for this study as this is an observational study.

Reporting for specific materials, systems and methods

We require information from authors about some types of materials, experimental systems and methods used in many studies. Here, indicate whether each material, system or method listed is relevant to your study. If you are not sure if a list item applies to your research, read the appropriate section before selecting a response.

Materials & experimental systems

n/a	Involved in the study	
☒	☐	Antibodies
☐	☒	Eukaryotic cell lines
☒	☐	Palaeontology and archaeology
☒	☐	Animals and other organisms
☐	☒	Human research participants
☒	☐	Clinical data
☒	☐	Dual use research of concern

Methods

n/a	Involved in the study	
☒	☐	ChIP-seq
☒	☐	Flow cytometry
☒	☐	MRI-based neuroimaging

Eukaryotic cell lines

Policy information about cell lines

Cell line source(s)	VeroE6/TMPRSS2 cells (JCRB1819)
Authentication	The cell line was obtained from JCRB cell bank of Okayama University; Cat#JCRB1819 in November 2020. We have not further authenticated this cell line afterwards
Mycoplasma contamination	The cell line has been recently tested negative for mycoplasma contamination in December 2021
Commonly misidentified lines (See ICLAC register)	No commonly misidentified cell were used.

Human research participants

Policy information about studies involving human research participants

Population characteristics	For the serosurveillance study, sampling and analyses were stratified by 10-year age groups (0-9 year to > or = 80 years). In this study, we did not record the sex of the patient cohorts. For the viral genomic part, respiratory specimens were collected

	from laboratory-confirmed cases in Hong Kong.
Recruitment	Specimens were collected for routine clinical purposes
Ethics oversight	This study was approved by the Institutional Review Board of the University of Hong Kong/Hospital Authority Hong Kong West Cluster (UW 21-131 and UW 18–141). Written informed consent was waived since archived anonymized specimens were used and did not include any personal or clinical information.

Note that full information on the approval of the study protocol must also be provided in the manuscript.
